# Association between lumbar disc herniation and facet joint osteoarthritis

**DOI:** 10.1186/s12891-020-3070-6

**Published:** 2020-01-29

**Authors:** Kai Zhu, Qihang Su, Tao Chen, Jinbiao Zhang, Mingjie Yang, Jie Pan, Weiping Wan, Aihong Zhang, Jun Tan

**Affiliations:** 1 Department of Orthopedics, Shanghai East Hospital, Tongji University School of Medicine, No.150 Jimo Road, Shanghai, 200120 China; 2 Department of Orthopedics, Shanghai Tongji Hospital, Tongji University School of Medicine, No.389 Xincun Road, Shanghai, 200092 China; 3 Department of Radiology, Changzheng Hospital, Second Military Medical University, Shanghai, 200003 China; 4 Department of Medical Statistics, Tongji University School of Medicine, No.1239 Siping Road, Shanghai, 200092 China; 5 Department of Orthopedics, Pinghu Second People’s Hospital, Pinghu, 314200 China

**Keywords:** Lumbar disc herniation, Facet joint osteoarthritis, Magnetic resonance imaging, Mixed-effects ordinal logistic regression model

## Abstract

**Background:**

This study was performed to investigate the association between lumbar disc herniation (LDH) and facet joint osteoarthritis (FJOA) using magnetic resonance imaging (MRI).

**Methods:**

Between March 2012 and September 2018, a total of 441 segments from 394 patients with LDH were included in the study. LDH was classified according to the Michigan State University (MSU) classification, in which the degree of LDH is divided into 3 levels (expressed as 1, 2, and 3) and the location of LDH is divided into 4 zones (described as A, AB, B, and C). Bilateral FJOA was graded from 0 to 3 using the criteria introduced by Weishaupt et al., and bilateral facet orientations were measured on axial MRI slices. A mixed-effects ordinal logistic regression model was utilized to determine the potential factors that may be associated with FJOA, including sex, age, body mass index (BMI), segment, facet orientation and tropism, and the degree and location of LDH.

**Results:**

In general, the prevalence of FJOA (grade ≥ 2) was 66.2% in LDH segments. For both the left and right sides, the degree of LDH was associated with the severity of FJOA (*p* < 0.01). Age and BMI were also associated with the severity of left and right FJOA (*p* = 0.002 and *p* < 0.001 for age, *p* < 0.001 and *p* = 0.003 for BMI, respectively), while segment, facet orientation, and facet tropism were not (*p* > 0.05 for all). Notably, MSU-B LDH was associated with greater odds of having more severe FJOA on the herniation side (left: *p* < 0.001, odds ratio (OR) = 2.714, 95% confidence interval (CI) = 1.583~4.650; right: *p* = 0.003, OR = 2.615, 95% CI = 1.405~4.870). However, other locations of LDH were not associated with the severity of FJOA (*p* > 0.05 for all).

**Conclusions:**

Both the degree of LDH and MSU-B LDH are associated with the severity of FJOA. The association between LDH and FJOA highlights the complexity of the etiology of FJOA.

## Background

Posterior paired facet joints (FJs) and anterior intervertebral discs constitute a spinal functional unit, which is termed the “three-joint complex”, indicating the intimate relationship between them [1, 2]. Osteoarthritis (OA) is the most common form of FJ pathology. According to statistics, the prevalence of lumbar FJOA, based on CT imaging, is 59.6% in men and 66.7% in women among the US adult population [3]. Another community-based study documented that the prevalence of lumbar FJOA was 17.58% among Korean adults aged 20 years and older [4]. However, knowledge about the etiology of FJOA remains very limited.

FJOA is considered a consequence of spinal degeneration as well as a potential source of low back pain [5]. Multiple studies have proposed several factors that are associated with FJOA, including age [6], body mass index (BMI) [7], facet orientation [8, 9], spinal and pelvic alignments [10], and disc degeneration [11]. Lumbar disc herniation (LDH) was defined as “a localized or focal displacement of disc material beyond the limits of the intervertebral disc space” [12]. Many existing studies have considered LDH as a radiologic manifestation or pathological consequence of disc degeneration [13–15]. However, recent evidence suggests that LDH may be clinically and radiologically different from disc degeneration [16]. Furthermore, the term “disc degeneration” is a broad and vague concept that can hardly describe the unique features of disc herniation. Although the correlation between disc degeneration and FJOA has been well established, little is known about the relationship between LDH and FJOA. From the clinical point of view, both LDH and FJOA are common causes of low back pain, and it is sometimes difficult to distinguish the exact source of clinical symptoms. Hence, it is reasonable to assume that there may be some connection between LDH and FJOA.

In the present study, we hypothesized that either the location or the degree of LDH may be associated with the severity of FJOA. The possible association between LDH and FJOA was investigated using magnetic resonance imaging (MRI).

## Methods

### Subjects

This was a retrospective cross-sectional study approved by Shanghai East Hospital (East Hospital Affiliated to Tongji University) Medical Ethics Committee (Shanghai, China). The clinical and radiographic data of 532 consecutive patients aged 18 to 60 years were reviewed. All patients came from the orthopedic clinic of Shanghai East Hospital between March 2012 and September 2018. They were diagnosed with LDH based on clinical presentations and radiological evidence including MRI. The diagnosis was made by Professor Jun Tan who has more than 20 years of experience in spine surgery. The diagnostic criteria include: 1. definite disc herniation shown on MRI; 2. back pain with or without radiculopathy and clinical signs that were consistent with radiographic findings [17]. Disc bulging was not included in this study. To minimize confounding variables that may affect the FJs, patients with the following diseases were excluded from this study: lumbar spondylolisthesis, scoliosis, severe central canal stenosis, and other congenital spinal deformities; and tumor, infection, rheumatoid arthritis, ankylosing spondylitis, and other systemic diseases. Additionally, patients with unsatisfactory imaging were also excluded from the study.

### Imaging evaluation

MRI (in the neutral supine position) (Intera Achieva 3.0 T; Philips Healthcare, Best, the Netherlands) was performed for all patients. MRI sequence parameters: sagittal fast spin echo T1-weighted imaging (FSE T1WI): field of view (FOV) =300 mm, repetition time/echo time (TR/TE) =550 ms/11 ms; sagittal FSE T2WI: FOV = 300 mm, TR/TE = 3000 ms/100 ms; axial FSE T2WI: FOV = 200 mm, TR/TE = 3500 ms/100 ms; slice thickness: 3–5 mm.

### LDH

**Fig. 1:**
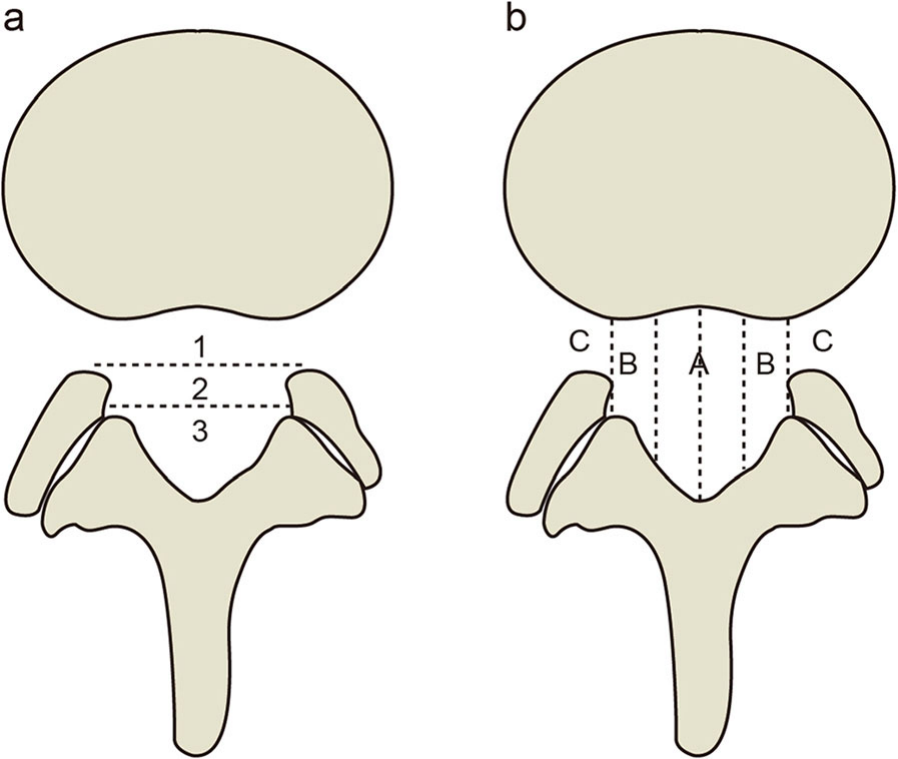
MSU classification for lumbar disc herniation. **a**: degree of disc herniation; **b**: location of disc herniation

The Michigan State University (MSU) classification [18] (Fig. 1) was used to evaluate LDH based on T2-weighted axial MRI slices. In this classification, the size of LDH is expressed as “1, 2, 3”, while the location of LDH is expressed as “A, AB, B, C”, which approximately corresponds to “central”, “paracentral”, “lateral” and “far lateral”. These subgrouping methods are based on an intra-facet line drawn transversely across the lumbar canal, to and from the medial edges of the right and left facet joint articulations. “1” and “2” are when the LDH extends less than or more than 50% of the distance from the non-herniated posterior aspect of the disc to the intra-facet line, and “3” is when the LDH extends beyond the intra-facet line. To define the location of the LDH, three points are placed along the intra-facet line, dividing it into four equal quarters; then, three vertical lines are drawn through these points, and four quadrants are created. “A” represents the left and right central quadrants, “B” represents the left and right lateral quadrants, “C” represents the area extending beyond the borderline of the lateral quadrants, and “AB” means that the furthest herniation is on the right and left lateral vertical lines. The level with the maximal herniation was selected for evaluation.

### FJOA

**Fig. 2:**
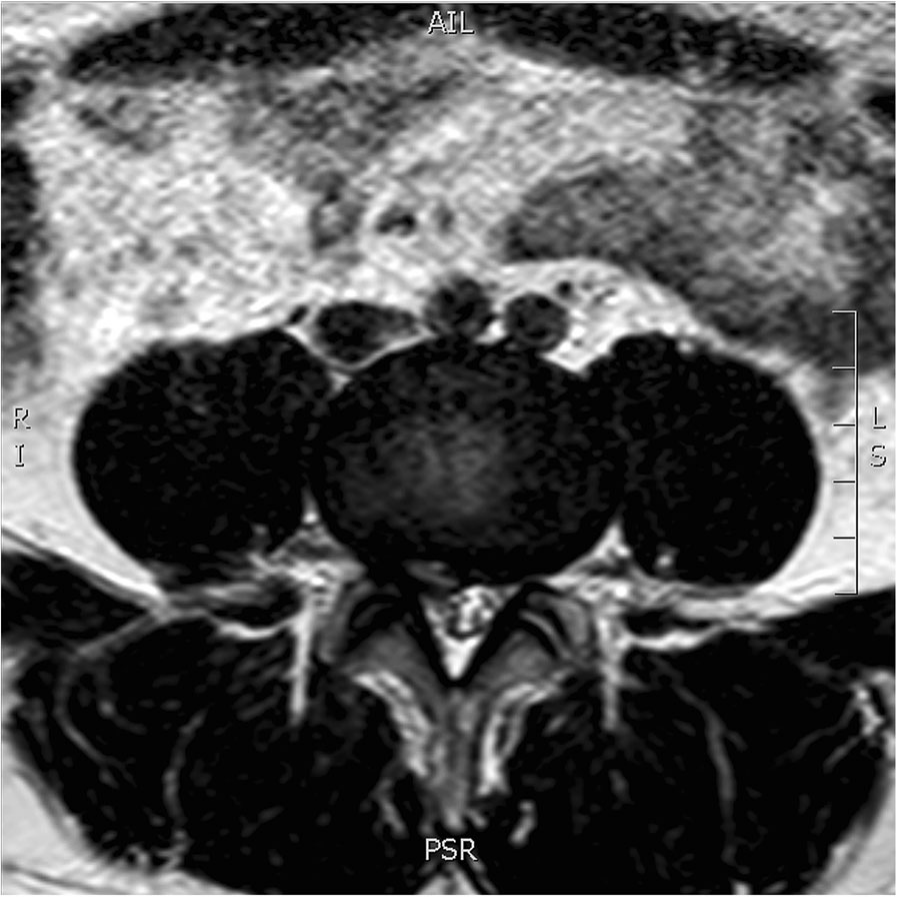
Axial T2-weighted MRI slice of a patient with LDH. LDH classification: MSU-2 (size) and right B (location); left FJOA: grade 0 (G0), and right FJOA: grade 2 (G2)

Right and left FJOA were graded separately using the criteria introduced by Weishaupt et al. [19] and Kalichman L et al. [9] (Fig. 2): grade 0 (G0): normal; grade 1 (G1): joint space narrowing (< 2 mm) and/or mild osteophytes and/or hypertrophy; grade 2 (G2): joint space narrowing (1 mm) and/or moderate osteophytes and/or moderate hypertrophy and/or subchondral erosions; grade 3 (G3): joint space narrowing (bone to bone) and/or severe osteophytes and/or severe hypertrophy and/or severe subchondral erosions and/or subchondral cysts. Grade ≥ 2 was considered substantial FJOA.

### FJ orientation and tropism

**Fig. 3:**
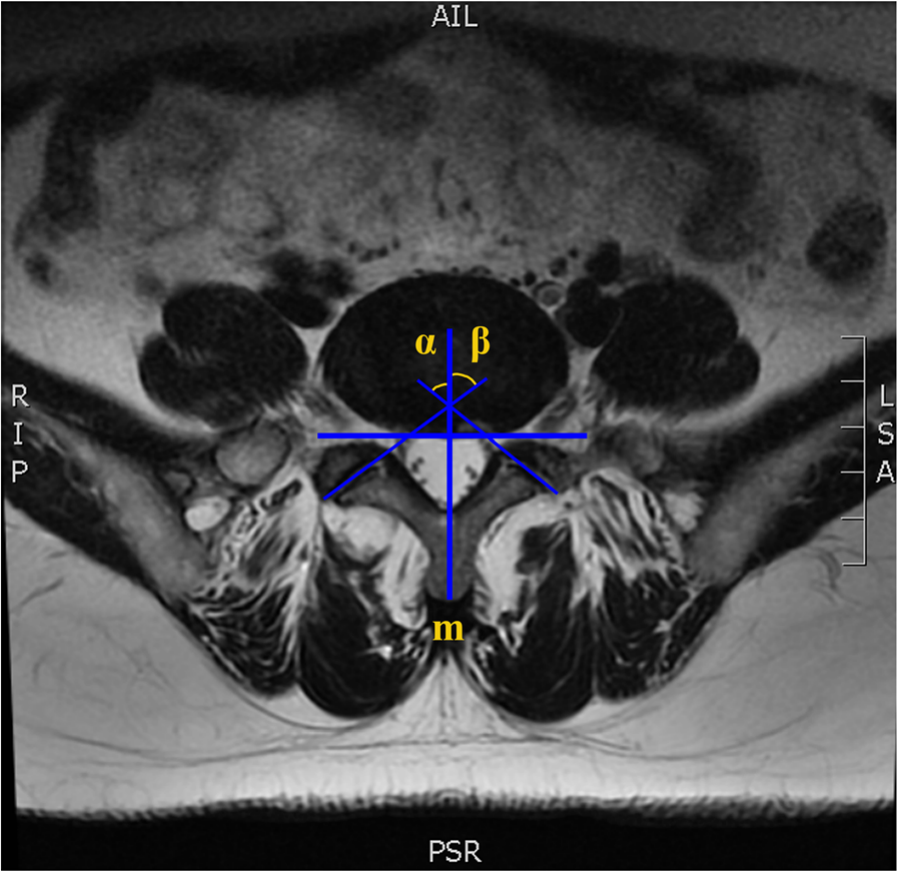
MRI measurement of facet joint orientation. m: middle line, a reference line bisecting the base of the spinous process; α: the angle between the middle line and the left facet line connecting the margins of the superior articular process;β: the right facet joint orientation

On an axial MRI slice that bisected the intervertebral disc, FJ angles relative to the sagittal plane were measured using the method described by Karacan I et al. [20] (Fig. 3). The FJ angle was defined as the angle between the reference line bisecting the base of the spinous process and the facet line connecting the margins of the superior articular process. Continuous FJ tropism was defined as the absolute difference between the left and right FJ angles.

### Reliability of the assessment

All the evaluations were performed primarily by an experienced spine specialist (MJY) who was blinded to the patients’ details and to the study hypothesis. Before the formal assessment, he was trained by a senior radiologist (WPW) who specialized in the musculoskeletal system. First, 40 LDH segments (80 FJs) were randomly selected for evaluation and the interexaminer reliabilities for the spine specialist and the radiologist were calculated. Then, the spine specialist read another 40 segments on two separate occasions, and the intraexaminer reliability was obtained. Finally, the weighted Kappa values for interexaminer reliability were 0.845 and 0.883 for FJOA and the degree of LDH, respectively; the Kappa value for interexaminer reliability was 0.808 for the location of LDH. The weighted Kappa values for intraexaminer reliability were 0.885 and 0.934 for FJOA and the degree of LDH, respectively; the Kappa value for intraexaminer reliability was 0.904 for the location of LDH. These data represent good to excellent reproducibility. The FJ angle was measured by the above two doctors, and the mean values were used.

### Statistical analysis

Because some patients in our study had multi-segment LDH and the data collected on those segments within one patient are not independent of each other, a mixed-effects ordinal logistic regression model was preferred to determine the potential factors associated with the severity of FJOA. Data analysis was conducted for the left and right sides, separately.

In this model, FJOA was treated as the response or dependent variable. The participant ID was used as random effect. Age, sex, BMI, level, facet tropism, facet orientation and LDH were treated as fixed effects. The difference between bilateral FJ angles can be classified into three types: left > right, right > left and left = right. All the LDH parameters were included in the same model. The cumulative logit function was used for ordinal response. An unstructured covariance structure was used for the statistical modeling. Statistical analysis was carried out using SPSS 23.0 software (SPSS Inc., Chicago, IL, USA); *P* < 0.05 was considered significant. The data are presented as the mean ± standard deviation.

## Results

**Fig. 4:**
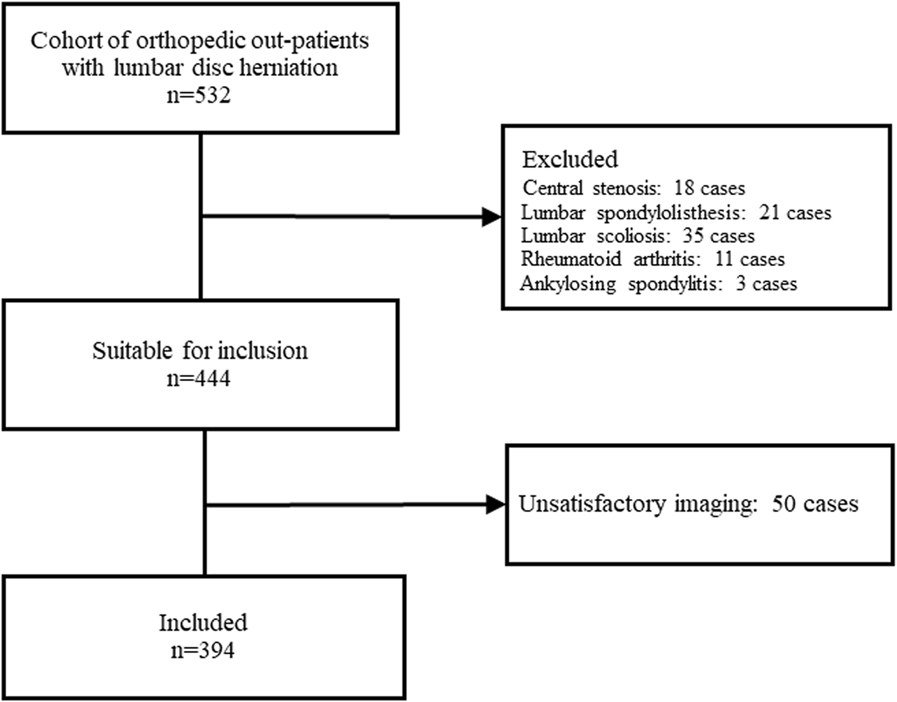
Flow chart diagram of patient selection

Finally, a total of 441 segments from 394 patients (219 males and 175 females) met the inclusion criteria. A flowchart of the patient selection process is shown in Fig. 4. The average age of patients was 43.5 ± 10.7 years old. The detailed clinical characteristics of the patients are shown in Table 1.

**Table 1:** Patient Demographics (*n* = 394)

Variables	
Gender(no.)	
Male (%)	219 (55.6)
Female (%)	175 (44.4)
Mean age (years)	43.5 ± 10.7
Segment of LDH(no.)	441
L2–3(%)	2 (0.5)
L3–4(%)	15 (3.4)
L4–5(%)	158 (35.8)
L5-S1 (%)	266 (60.3)
BMI (kg/m^2^)	23.9 ± 2.8
Facet tropism (°)	6.3 ± 5.3

*BMI* body mass index, *LDH* lumbar disc herniation

### Prevalence of FJOA in LDH patients

In general, the prevalence of FJOA (grade ≥ 2) was 66.2% in LDH segments. Specifically, the prevalence of FJOA was 56.3, 71.1, and 72.1% in MSU-1, MSU-2, and MSU-3 LDH segments, respectively, and 60.9, 68, 74, and 65.9% in MSU-A, MSU-AB, MSU-B, and MSU-C LDH segments, respectively (Table 2).

**Table 2:** Distribution of FJOA in LDH patients (441 segments, 882 FJs)

LDH	FJOA				Total
	G0	G1	G2	G3	
Degree of LDH					
MSU-1	24 (8.0%)	107 (35.7%)	138 (46.0%)	31 (10.3%)	300
MSU-2	13 (2.9%)	116 (26.0%)	254 (57.0%)	63 (14.1%)	446
MSU-3	3 (2.2%)	35 (25.7%)	58 (42.7%)	40 (29.4%)	136
Total	40 (4.5%)	258 (29.3%)	450 (51.0%)	134 (15.2%)	882
Location of LDH					
MSU-A	26 (6.2%)	138 (39.9%)	206 (49.0%)	50 (11.9%)	420
MSU-AB	9 (5.2%)	46 (26.7%)	95 (55.2%)	22 (12.8%)	172
MSU-B	5 (2.0%)	59 (24.0%)	126 (51.2%)	56 (22.8%)	246
MSU-C	0 (0.0%)	15 (34.1%)	23 (52.3%)	6 (13.6%)	44
Total	40 (4.5%)	258 (29.3%)	450 (51.0%)	134 (15.2%)	882

*FJOA* facet joint osteoarthritis, *FJs* facet joints, *LDH* lumbar disc herniation, *MSU* Michigan State University

### Association between LDH and the severity of FJOA

For both sides, the degree of LDH was associated with the severity of FJOA (*p* < 0.01). Aging and BMI were also associated with the severity of left and right FJOA (*p* = 0.002 and *p* < 0.001 for age, *p* < 0.001 and *p* = 0.003 for BMI, respectively), while segment, facet orientation, and facet tropism were not (*p* > 0.05 for all). Notably, MSU-B LDH was associated with greater odds of having more severe FJOA on the herniation side (Left: *p* < 0.001, odds ratio (OR) =2.714, 95% confidence interval (CI) = 1.583~4.650; Right: *p* = 0.003, OR = 2.615, 95% CI = 1.405~4.870). However, other locations of LDH were not associated with the severity of FJOA (*p* > 0.05 for all). The results of GLMM analysis are summarized in Table 3.

**Table 3:** Multivariable generalized linear mixed model describing the potential factors associated with FJOA

Variables	FJOA(L)		FJOA(R)	
	*P*	OR(95%CI)	*P*	OR(95%CI)
Gender				
Female	–	–	–	–
Male	0.372	1.191 (0.811~1.747)	0.778	1.057 (0.721~1.549)
Age	0.002^*^	1.028 (1.010~1.046)	0.000^*^	1.034 (1.016~1.052)
Facet tropism	0.754	0.994 (0.959~1.031)	0.276	0.980 (0.946~1.016)
BMI	0.000^*^	1.136 (1.063~1.214)	0.003^*^	1.105 (1.035~1.180)
Levels				
L2/3	0.408	0.327 (0.023~4.654)	0.109	0.113 (0.008~1.629)
L3/4	0.935	1.047 (0.351~3.119)	0.718	1.223 (0.409~3.650)
L4/5	0.199	0.773 (0.522~1.146)	0.065	0.691 (0.467~1.023)
L5/S1	–	–	–	–
LDH degree				
MSU-1	–	–	–	–
MSU-2	0.009^*^	1.731 (1.144~2.619)	0.090	1.427 (0.946~2.152)
MSU-3	0.001^*^	2.590 (1.446~4.640)	0.000^*^	3.258 (1.810~5.865)
LDH location				
A	–	–	–	–
AB(L)	0.243	0.680 (0.355~1.300)	0.817	1.080 (0.563~2.071)
B(L)	0.000^*^	2.714 (1.583~4.650)	0.956	0.986 (0.585~1.661)
C(L)	0.370	1.594 (0.574~4.427)	0.723	1.200 (0.437~3.297)
AB(R)	0.788	0.918 (0.492~1.714)	0.660	1.150 (0.616~2.146)
B (R)	0.652	1.150 (0.624~2.119)	0.003^*^	2.615 (1.405~4.870)
C(R)	0.462	0.554 (0.115~2.678)	0.890	0.895 (0.184~4.354)
Facet angles				
L = R	–	–	–	–
L > R	0.847	1.090 (0.452~2.631)	0.232	1.705 (0.710~4.093)
L < R	0.560	1.294 (0.543~3.084)	0.273	1.619 (0.683~3.834)

* *p* < 0.05

- reference

*BMI* body mass index, *CI* confidence interval, *FJOA* facet joint osteoarthritis, *L* left, *LDH* lumbar disc herniation, *MSU* Michigan State University, *R* right, *OR* odds ratio

## Discussion

To the best of our knowledge, this is the first study to investigate the association of parameters of LDH and FJOA. Previous studies on FJOA seemed to ignore the difference in severity between left and right FJOA at the same vertebral level. For instance, when evaluating asymmetric FJOA in one motion segment, some researchers used the more serious grade as the representative degree of FJOA of the segment [10, 21, 22]. However, asymmetric FJOA is a common radiographic demonstration that should not be ignored [23]. Hence, for a more fair and accurate evaluation of FJOA, we investigated and analyzed the two sides of the FJ separately. We found that both the location and degree of LDH were associated with the severity of FJOA. Patients with MSU-B LDH have greater odds of having more severe FJOA on the herniation side. This finding may expand the current understanding of the etiology of FJOA. From a clinical point of view, it has been reported that severe FJOA was significantly associated with frequent back pain [5]. Hence, doctors need to pay more attention to the FJOA on the herniation side when treating MSU-B LDH.

Our results demonstrated that MSU-B LDH was associated with more severe FJOA. There are two possible hypotheses to explain this. First, MSU-B LDH is closer to the ipsilateral FJ articulations than other locations of LDH, which may facilitate the stimulation of disc fragments to the adjacent FJ tissues. As indicated by Igarashi A, et al. [24], the inflammatory factors produced in FJ tissues may leak into the intraspinal space via the lateral part of the ventral facet joint capsule. Hence, it is reasonable to hypothesize that the inflammatory factors and chemical substances released from the herniated disc could leak into the FJ tissues and may affect the severity of FJOA. Additionally, in MSU-A and MSU-AB cases, the existence of the posterior longitudinal ligament may help to impede the diffusion of the disc-derived stimulators. Second, from a biomechanical point of view, MSU-B LDH is more likely to cause asymmetry in bilateral disc space narrowing when compared with MSU-A and MSU-AB, and this may increase the stress of the FJ on the narrower side and promote the ipsilateral FJOA [25]. Under this assumption, the reason why MSU-C LDH was not associated with the severity of FJOA is possibly due to the small sample size with this type in our study.

The above hypotheses can also explain the association between the degree of LDH and the severity of FJOA. A more protruded disc is likely associated with a greater loss of intervertebral height, which is a risk factor for FJ degeneration due to increased FJ loading [26]. In this study, we observed that a more protruded disc was usually combined with the rupture of the outer annulus fibrosus (AF), which may facilitate the release of disc materials and affect the adjacent FJ. In contrast, small-sized LDH was found to have a relatively intact outer AF. We also noticed that MSU-2 LDH was significantly associated with the severity of the left FJOA, but not the right FJOA. We believe that this differential effect was caused by the difference in severity of left and right FJOA. To prove this, we performed a hypothesis test. The results (Additional file 1) demonstrated that in cases with MSU-2 LDH, the severity of the left FJOA was significantly higher than that of the right FJOA. In cases with MSU-3, no such difference was found.

It has been reported that the sagittal orientation of FJ may affect FJOA [8, 9]. Therefore, the difference in the severity of FJOA between sides could result from the difference in FJ orientation between sides. To eliminate this confounding factor, we measured and compared the FJ angle between sides. According to our results, MSU-B LDH was still significantly associated with the severity of FJOA after correction for the FJ angle. Additionally, sex, level and FJ tropism were not associated with the severity of FJOA, which is consistent with a previous study [9].

The major limitation of this study is the lack of a control group. We cannot determine whether patients with LDH are more likely to have FJOA than those without LDH.

Another limitation is that this study was based on MRI findings. We did not have clinical or pathological evidence. Based on our hypotheses, future work should be performed to validate the inflammatory mechanism by investigating the herniated disc and FJ tissues harvested from the surgery.

## Conclusion

Both the degree and the location (MSU-B) of LDH are associated with the severity of FJOA. This association highlights the complexity of the etiology of FJOA. Doctors should pay more attention to distinguish the source of clinical symptoms produced by severe FJOA and by MSU-B LDH.

## Supplementary information

**Additional file 1.** Hypothesis test for the distribution of the severity of bilateral FJOA and results for the unadjusted logistic regression models.

## Data Availability

The raw data are available upon reasonable request from the corresponding author (Jun Tan).

## Abbreviations

AF: Annulus fibrosus
BMI: Body mass index
FJOA: Facet joint osteoarthritis
LDH: Lumbar disc herniation
MRI: Magnetic resonance imaging
MSU: Michigan State University
