# Supplementary material for: Association between lumbar disc herniation and facet joint osteoarthritis
Source: BMC Musculoskelet Disord. 2020 Jan 29;21:56. doi: 10.1186/s12891-020-3070-6 (PMC6990568; doi:10.1186/s12891-020-3070-6)
Supplement: Supplementary file 1 — Additional file 1. Hypothesis test for the distribution of the severity of bilateral FJOA and results for the unadjusted logistic regression models. [file 12891_2020_3070_MOESM1_ESM.docx]

**Hypothesis test for the distribution of the severity of bilateral FJOA**


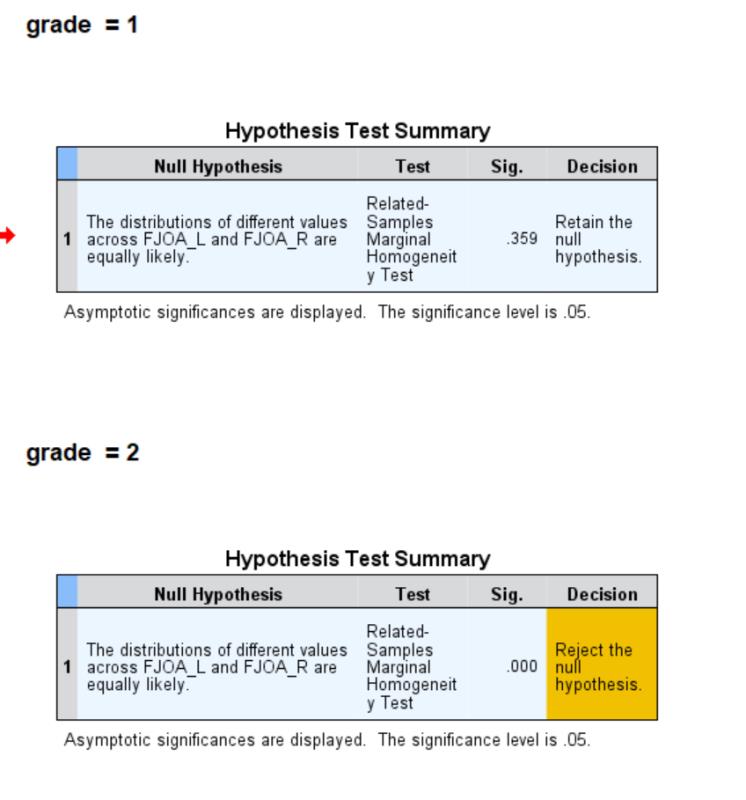


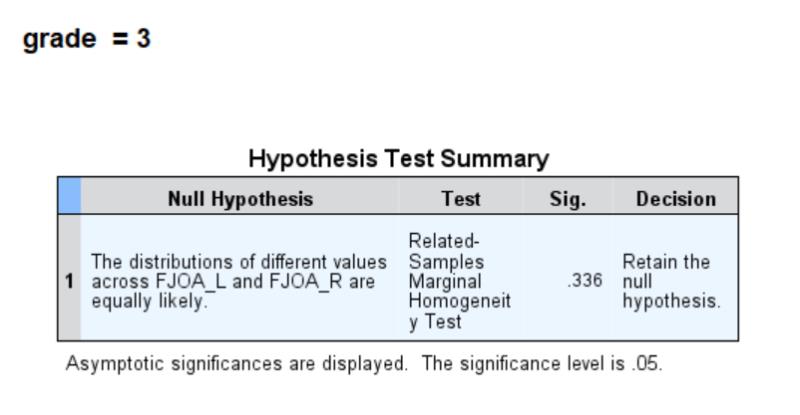


**Mixed-eﬀects ordinal logistic regression model: unadjusted**

| Variables | FJOA(L) | | | | |
| --- | --- | --- | --- | --- | --- |
|  |  |  | 95%CI | |  |
|  | β | OR | lower | upper | P |
| LDH degree |  |  |  |  |  |
| MSU-1 |  |  |  |  |  |
| MSU-2 | 0.55 | 1.733 | 1.162 | 2.585 | 0.007 |
| MSU-3 | 0.925 | 2.522 | 1.435 | 4.433 | 0.002 |
| LDH zone |  |  |  |  |  |
| A |  |  |  |  |  |
| AB(L) | -0.231 | 0.794 | 0.042 | 1.498 | 0.475 |
| B(L) | 1.12 | 3.064 | 1.821 | 5.156 | ＜0.001 |
| C(L) | 0.552 | 1.736 | 0.64 | 4.708 | 0.278 |
| AB(R) | -0.027 | 0.974 | 0.526 | 1.804 | 0.932 |
| B(R) | 0.304 | 1.355 | 0.746 | 2.463 | 0.318 |
| C(R) | 0.034 | 1.035 | 0.251 | 4.268 | 0.962 |

CI, confidence interval; FJOA, facet joint osteoarthritis; L, left; LDH, lumbar disc herniation; MSU, Michigan State University; R, right; OR, odds ratio

MSU-1 and A were treated as reference group

| Variables | FJOA(R) | | | | | |
| --- | --- | --- | --- | --- | --- | --- |
|  |  |  | 95%CI | |  | |
|  | β | OR | lower | upper | P | |
| LDH degree |  |  |  |  |  | |
| MSU-1 |  |  |  |  |  | |
| MUS-2 | 0.356 | 1.428 | 0.961 | 2.123 | 0.078 | |
| MSU-3 | 1.14 | 3.126 | 1.772 | 5.514 | <0.001 | |
| LDH zone |  |  |  |  |  | |
| A |  |  |  |  |  | |
| AB(L) | 0.227 | 1.255 | 0.664 | 2.374 | 0.484 | |
| B(L) | 0.054 | 1.056 | 0.639 | 1.743 | 0.832 | |
| C(L) | 0.192 | 1.211 | 0.452 | 3.245 | 0.703 | |
| AB(R) | 0.174 | 1.19 | 0.643 | 2.203 | 0.579 |  |
| B(R) | 1.012 | 2.751 | 1.499 | 5.046 | 0.001 | |
| C(R) | 0.634 | 1.886 | 0.452 | 7.876 | 0.383 | |

CI, confidence interval; FJOA, facet joint osteoarthritis; L, left; LDH, lumbar disc herniation; MSU, Michigan State University; R, right; OR, odds ratio

MSU-1 and A were treated as reference group
